# Plasma levels of immunosuppressive mediators during cardiopulmonary bypass

**DOI:** 10.1155/S0962935196000099

**Published:** 1996-02

**Authors:** E. Borrelli, P. Giomarelli, A. Naldini, E. Luzzi, S. Silvestri, M. Gardinali, V. Bocci

**Affiliations:** 1 Institute of Thoracic and Cardiovascular Surgery Via Laterina University of Siena Siena 8 - 53100 Siena Italy; 2 Institute of General Physiology Via Laterina University of Siena Siena 8 - 53100 Siena Italy; 3 2nd Medical Clinic University of Milan Milan Italy

## Abstract

The aim of this study was to evaluate plasma levels of two mediators with immunosuppressive properties, complement fraction C3a (C3a) and transforming growth factor-β_1_ (TGF-β_1_), during extracorporeal circulation. The proliferation index after phytohaemagglutinin (PHA) stimulation of isolated peripheral blood mononuclear cells was also investigated. Sixteen patients undergoing hypothermic (*n* = 8, group 1) and normothermic (*n* = 8, group 2) cardiopulmormry bypass (CPB) were enrolled in this study. As a control, we evaluated four patients undergoing thoracovascular operations without CPB. Blood samples were collected before CPB but after anaesthesia, every 30 min during CPB, at the end of CPB and 10 min after protamine administration. Both C3a and TGF-β_1_ increased significantly during CPB and after protamine administration in the hypothermic as well as the normothermic group. In the latter case the increase of C3a and TGF-β_1_, although more prominent, was not significantl higher than in the former group. Conversely, the proliferation, index of peripheral mononuclear cells had already decreased 30 min after CPB was started and remained depressed throughout the CPB time. These results suggest a possible role of C3a and TGF-β_1_ in the immunological changes occurring during extracorporeal circulation.


Mediators of Inflammation **5**, 51–55 (1996)

### Plasma levels of immunosuppressive mediators during cardiopulmonary bypass

E. Borrelli,[1] P. Giomarelli,[1] A. Naldini,[2] E. Luzzi,[2] S. Silvestri,[2] M. Gardinali[3] and V. Bocci[2,CA]

[1]Institute of Thoracic and Cardiovascular Surgery, and [2]Institute of General Physiology, Via Laterina, 8 - 53100 Siena, University of Siena, Siena. Fax: (+39) 577 282098; [3]2nd Medical Clinic, University of Milan, Milan, Italy.

[CA]Corresponding Author

**The aim of this study was to evaluate plasma levels of two mediators with immunosuppressive properties, complement fraction C3a (C3a) and transforming growth factor-β1 (TGF-β1), during extracorporeal circulation. The proliferation index after phytohaemagglutinin (PHA) stimulation of isolated peripheral blood mononuclear cells was also investigated. Sixteen patients undergoing hypothermic ($n = 8$, group 1) and normothermic ($n = 8$, group 2) cardiopulmonary bypass (CPB) were enrolled in this study. As a control, we evaluated four patients undergoing thoracovascular operations without CPB. Blood samples were collected before CPB but after anaesthesia, every 30 min during CPB, at the end of CPB and 10 min after protamine administration. Both C3a and TGF-β1 increased significantly during CPB and after protamine administration in the hypothermic as well as the normothermic group. In the latter case the increase of C3a and TGF-β1, although more prominent, was not significantly higher than in the former group. Conversely, the proliferation index of peripheral mononuclear cells had already decreased 30 min after CPB was started and remained depressed throughout the CPB time. These results suggest a possible role of C3a and TGF-β1 in the immunological changes occurring during extracorporeal circulation.**

**Key words:** Cardiopulmonary bypass, Complement, Hypothermia, Normothermia, Transforming growth factor-β

### Introduction

Current knowledge on cardiopulmonary bypass (CPB) suggests that complement activation and release of inflammatory mediators from activated platelets and polymorphonucleates into the bloodstream, is one of the most important pathophysiological changes occurring during extracorporeal circulation, and probably is responsible for a number of postoperative derangements such as the increase of capillary permeability, systemic vasodilatation and multiple organ dysfunction.[1–3]

Recently, it has been reported that the mediators released during CPB may also play a determinant role in the biochemical and clinical events, leading to the postoperative immunosuppression in cardiac surgery patients throughout a functional and quantitative decrease of T- and B-lymphocytes.[4] Experimental investigations showed that T-helper number, mitogen responsiveness and interleukin-2 production significantly decreased on the first postoperative day and remained depressed even on day 7.[5] Although high levels of cortisol may be a cause of the impairment of the immune system with a redistribution of circulating lymphocytes to lymphoid organs and bone marrow,[6] other inhibitory factors may be released during the CPB technique.

As suggested by our previous study,[7] using isolated T-lymphocytes, an inhibition of proliferation and cytokine production appears 30 min after the onset of CPB. To evaluate the causes of this early immunological defect during CPB, we have focused our attention on the plasma levels of two mediators with immunosuppressive effects, complement fraction C3a (C3a), and transforming growth factor-β1 (TGF-β1).

It is known that C3a, a soluble fragment derived from complement, suppresses antigen-induced T-cell proliferation, antibody production, T-cell and natural killer (NK) cell mediated cytotoxicity, as reported by studies *in vitro* and *in vivo*.[8] TGF-β1, a cytokine mainly released from the alpha granules of the activated platelets, is a multifunctional mediator that affects cell growth and differentiation, immunoresponses, angio-

© 1996 Rapid Science Publishers 

genesis and tissue repair.[9,10] The findings that receptors for TGF-β1 are ubiquitous further suggests that this substance may have an important role as a regulatory molecule. No data have been reported so far on its presence during CPB.

The aim of this study was to determine whether CPB was associated with a change of C3a and TGF-β1 plasma concentration and possibly with the decrease of *in vitro* T-cell proliferation. In recent years, a number of investigators have suggested the superiority of normothermic heart surgery compared to hypothermic heart surgery to reduce the adverse consequences of intraoperative myocardial ischaemia and cardiopulmonary bypass. Both approaches have shown advantages and disadvantages, and data are not conclusive.[11] To evaluate the possible differences in the activation of the inflammatory system, we have performed our study in normothermic (35°–37°C) and hypothermic (30°C) CPB.

### Materials and Methods

Sixteen patients undergoing extracorporeal circulation for coronary artery bypass disease were enrolled in this study. The patients were randomized in two groups: group 1 (eight patients) received hypothermic CPB and group 2 (eight patients) received normothermic CPB. There was no significant difference between the two groups with respect to the age of the patients and CPB times. The patients' characteristics are reported in Table 1. As a control, blood samples were drawn at the indicated times after anaesthesia in four patients undergoing thoracovascular operation without CPB. The influence of anaesthesia was eliminated by taking the baseline value after its induction, just before CPB or thoracovascular operation. It must be mentioned that, for ethical reasons, we could not check the influence of protamine in these controls.

Anaesthesia was uniform in all cases and considered of a standard combination of fentanyl citrate, diazepam, pancuronium bromide and isoflurane. CPB equipment consisted of a nonpulsatile roller pump, a membrane oxygenator and an arterial filter. Myocardial protection was performed in group 1 with anterograde cold blood cardioplegic solution as described by Buckberg,[12] and in group 2 with continuous intermittent normothermic blood cardioplegia as reported by Lichtenstein.[13] In patients undergoing cold CPB, the temperature of the circulating blood was cooled to 30°C, whereas those in the warm group were kept between 35° and 37°C. Pump flows were maintained at 2.5 l/min/m$^2$ in normothermic CPB, and about 2.2 l/min/m$^2$ in hypothermic CPB. Administration of heparin before cannulation and subsequent neutralization after bypass with protamine sulfate were performed in a standard fashion.

Whole-blood samples (5 ml) for C3a and TGF-β1 determination were drawn before CPB (T1) but after anaesthesia at 30 (T2), 60 (T3) and 90 (T4) min during CPB, immediately before the end of CPB (T5), and 10 min after protamine administration (T6). After platelets count, blood–EDTA was immediately centrifuged and the plasma was stored at −80°C until assayed.

C3a was measured in the plasma samples by radioimmunoassay[14] and corrected for haemodilution (haematocrit of sample/haematocrit before CPB × 100). Data were expressed as ng/ml.

TGF-β1 (expressed as ng/ml of plasma) was measured by ELISA by using the method and the reagents kindly provided by Dr W. Waegell of Celtrix Laboratories Inc., Palo Alto, CA, USA. In this preliminary investigation we have not attempted to measure the TGF-β in a latent form[9,15] so that reported values are only for the biologically mature form. All plasma samples were diluted at least 1:1 with the appropriate diluent. A three-cycle automatic washing was routinely performed. Negative plasma samples, in the absence or presence of haemoglobin, were spiked with the cytokine standards to assess the reliability and precision of the various assays. Yields ranged between 86 and 107%.

For *in vitro* T-cell isolation, samples of venous blood (10 ml) were obtained from each patient of both groups at the following times: before the institution of CPB, at 30 and 60 min during CPB and at the end of CPB.

After a gradient of Lymphoprep and centrifugation, peripheral blood mononuclear cells (PBMC) were removed, resuspended in medium and incubated, following the standard procedure. The proliferative response of PBMC to phytohaemagglutinin (PHA) was determined using the colorimetric method described by Mosmann,[16] based on the tetrazolium salt 3-(4,5 dimethylthiazol-2-yl)-2,5-diphenyltetrazolium bromide (MTT). Briefly, 2 × 10$^5$ cells/well were incubated in 200 μl of culture medium in the presence or absence of PHA for 40 h. After removing 100 μl

**Table 1.** Patients' characteristics

| Characteristic | Hypothermic | Normothermic |
|---|---|---|
| Age (Y) | 62 ± 4 | 64 ± 4 |
| Sex (M:F) | 6:2 | 5:3 |
| Grafts (NR) | 3 ± 1 | 3 ± 1 |
| CPB time | 110 ± 10 | 100 ± 6 |

of medium, 10 µl of a solution of MTT (5 mg/ml) was added to each well and incubated at 37°C. After 4 h 100 µl of acid propan-2-ol (0.04 M HCl in propan-2-ol) was added to each well and after all the formazan crystals had dissolved, the plate was read on a microelisa reader. The optical density values (O.D.) were obtained, using a text wavelength of 570 nm and a reference wavelength of 630 nm. The proliferation index (PI), used to quantitate the PBMC response to PHA, was calculated as PI = PHA treated lymphocyte activity (O.D.)/untreated lymphocyte activity (O.D.).

*Statistical analysis:* Data were expressed as the mean ± S.E.M. For intragroup analysis, a Student's *t*-test for paired data was used. Intergroup differences were estimated by the ANOVA test. $p < 0.05$ was assumed to be significant.

### Results

Thirty min after the start of CPB (T2), the plasma level of C3a increased from its baseline value in both groups (2621 ± 355 ng/ml *vs.* 287 ± 37 ng/ml, group 1, hypothermia; 2632 ± 626 ng/ml *vs.* 262 ± 18 ng/ml, group 2, normothermia; $p < 0.01$), and it remained high during the CPB period (Fig. 1). In the controls without CPB the plasma level of C3a increased towards the end of the operation but it tended to normalize quickly (Table 2). After protamine administration, C3a rose in both normothermic and hypothermic CPB (2875 ± 506 ng/ml, group 1; 3275 ± 684 ng/ml, group 2). Although normothermic C3a values were higher than hypothermic ones, no significant intergroup differences were evident.

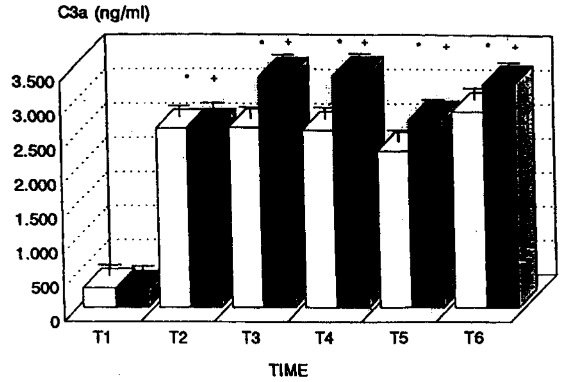

FIG. 1. Time course of C3a plasma levels during hypothermic (white bars) and normothermic (black bars) cardiopulmonary bypass. T1 = before CPB and after anaesthesia; T2 = 30 min after CPB was started; T3 = 60 min after CPB was started; T4 = 90 min after CPB was started; T5 = immediately before CPB end; T6 = 10 min after protamine administration. * $p < 0.05$ *vs.* T1, group 1; $^{+}p < 0.05$ *vs.* T1, group 2. □, hypothermic CPB; ■, normothermic CPB.

TGF-β1 increased significantly after 30 min of extracorporeal circulation (6.24 ± 1.75 *vs.* 1.6 ± 0.23, group 1; 9.73 ± 2.14 ng/ml *vs.* 2.35 ± 0.36 ng/ml, group 2, $p < 0.05$). Also in this case, high levels of TGF-β1 persisted until CPB ended in both groups. No change of TGF-β1 plasma levels was noted in the control group (Table 2). Unexpectedly, peak values of TGF-β1 were reached 10 min after protamine administration (25.93 ± 6.39 ng/ml, group 1; 39.2 ± 5.44 ng/ml, group 2). TGF-β1 in normothermic extracorporeal circulation was consistently higher than in hypothermic CPB (Fig. 2). However the difference was not statistically significant.

Platelet numbers (×1,000) showed a fall 30 min after the onset of CPB in both groups (161 ± 18 *vs.* 276 ± 29, group 1; 167 ± 35 *vs.* 242 ± 32, group 2) not observed in the controls. There was a slight increase during CPB, followed by a decreased level after protamine administration (Fig. 3). Without CPB (control group) platelet levels did not vary (Table 2).

To evaluate the effect of CPB on cell proliferation, PBMC were isolated from blood samples during CPB and a similar number of cells were cultured in the presence or absence of PHA (5 µg/ml). At variance with the controls, the proliferation index (PI) was greatly affected during CPB, as shown in Fig. 4. PI significantly decreased as soon as 30 min after CPB commenced (PI at 30 min, 1.26 ± 0.08 *vs.* 1.59 ± 0.09 of baseline value, group 1; PI at 30 min, 1.36 ± 0.04 *vs.* 1.74 ± 0.06 of baseline value, group 2; $p < 0.01$); at the end of CPB the PI decreased further (1.23 ± 0.05 group 1, $p < 0.05$; 1.58 ± 0.06 group 2, $p < 0.05$). There was also a slight, but not significant decrease of

**Table 2.** Values with mean ± S.E.M. of C3A, TGF-β1, platelets and proliferation index obtained from four control samples undergoing a thoracovascular operation

| Time | C3a (ng/ml) | TGF-β (ng/ml) | PLT (×1,000) | PI(MTT) |
|---|---|---|---|---|
| T1 (after anaesthesia) | 200 ± 30 | 1.37 ± 0.27 | 270 ± 33 | 1.48 ± 0.22 |
| T5 (before the end of the operation) | 610 ± 100 | 1.47 ± 0.15 | 256 ± 28 | 1.34 ± 0.19 |
| T6 (30 min after operation) | 345 ± 86 | 1.35 ± 0.18 | 262 ± 42 | 1.36 ± 0.25 |

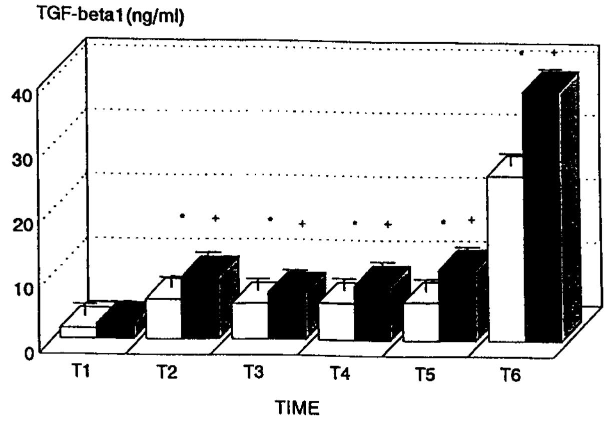

FIG. 2. Time course of TGF-β1 plasma levels during hypothermic (white bars) and normothermic (black bars) CPB. T1 = before CPB; T2 = 30 min after CPB was started; T3 = 60 min after CPB was started; T4 = 90 min after CPB was started; T5 = immediately before CPB end; T6 = 10 min after protamine administration. *$p < 0.05$ *vs.* T1, group 1; $^{+}p < 0.05$ *vs.* T1, group 2. Key as in Fig. 1.

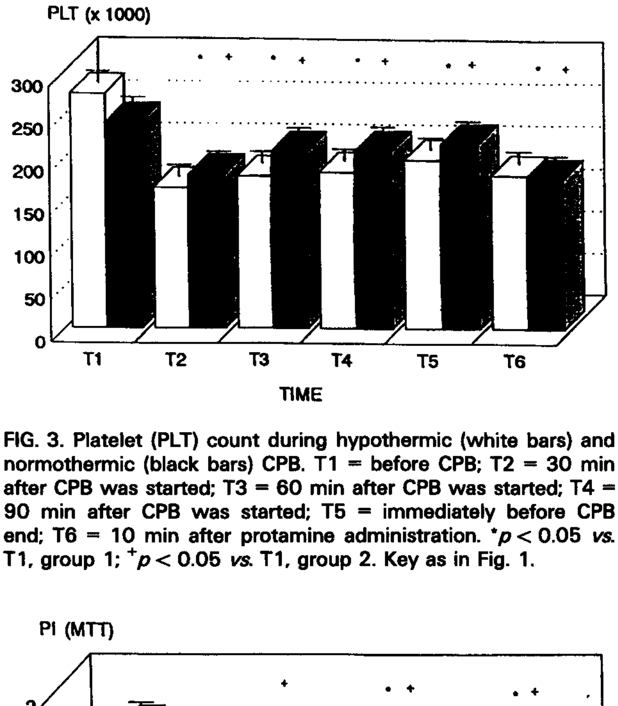

FIG. 3. Platelet (PLT) count during hypothermic (white bars) and normothermic (black bars) CPB. T1 = before CPB; T2 = 30 min after CPB was started; T3 = 60 min after CPB was started; T4 = 90 min after CPB was started; T5 = immediately before CPB end; T6 = 10 min after protamine administration. *$p < 0.05$ *vs.* T1, group 1; $^{+}p < 0.05$ *vs.* T1, group 2. Key as in Fig. 1.

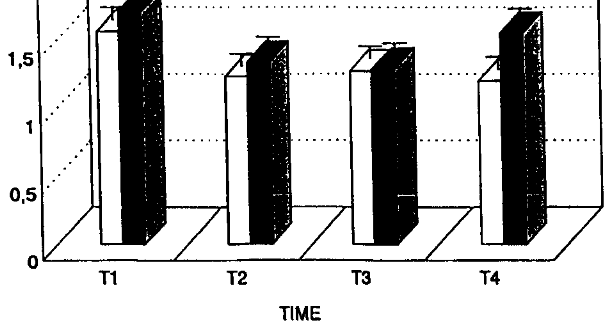

FIG. 4. Proliferation index of circulating lymphocytes after isolation and PHA stimulation during hypothermic (white bars) and normothermic (black bars) CPB. T1 = before CPB; T2 = 30 min after CPB was started; T3 = 60 min after CPB was started; T4 = immediately before CPB end. *$p < 0.05$ *vs.* T1, group 1; $^{+}p < 0.05$ *vs.* T1, group 2. Key as in Fig. 1.

the PI during the thoracovascular operation (Table 2).

### Discussion

CPB causes a number of inflammatory and immunologic responses, documented in a series of studies,[1–3] by the increased plasma levels of several compounds which are not readily correlated with the physiopathological modifications. Clinical observation on the increased susceptibility to infections in postoperative cardiac patients has suggested a CPB related T- and B-cell immunodeficiency.[17] Profound lymphopenia with drastic reduction in T-helper-lymphocytes was reported from 24 h to 3–4 days after operation,[17] and other investigators found changes in the CD4/CD8 ratio as early as 2 h after CPB. Our recent study,[7] performed during CPB, showed that lymphocyte function was already suppressed 30 min after CPB had commenced, and remained throughout the CPB time. This interesting experimental finding suggests that the cause of the immunosuppression could be due to very early events mainly occurring during extracorporeal circulation.

It is well known that the complement system is rapidly activated through the contact of blood with foreign materials of the oxygenator,[3] and recently it has been reported[8,18,19] that complement by-products play an important modulatory role in the inductive phase of the immune response, throughout a cooperation between immobilized C3 split products (that stimulate T- and B-cell activation) and the soluble C3a product, which inhibit lymphocyte proliferation. Moreover, other studies have shown that soluble complement fragments generated during decomplementation may be immunosuppressive.[8] Thus, the high levels of C3a during CPB determined in the present study (Fig. 1) agree with the possibility of an immunosuppressive effect.

Perhaps the best known effect of TGF-β1 is on the cells of the immune system: TGF-β1 inhibits T- and B-cell proliferation without affecting IL-2 or IL-2 receptor expression, and also inhibits NK cell activity.[20,21] Data reported in our study indicate that during extracorporeal circulation, the decrease of platelets and their degranulation is concomitant with the release of TGF-β1 from the alpha granules, with its consequent increase in the bloodstream. Because TGF-β1 mediates the immunodepressive effects at levels in the fentomolar ranges, it appears reasonable to postulate that binding of TGF-β1 to circulating immune cells may lead later to their hyporesponsiveness to PHA, as shown by the decrease of the PI in Fig. 4. Furthermore, it is well known that protamine administration causes an activation of the classic pathway of the complement system.[18] The concomitant increase of circulating levels of C3a and TGF-β1 and the fall of platelet count, 10 min after protamine administration, are presumably linked to complement changes and platelet degranulation.

As far as the comparison between normothermic and hypothermic CPB is concerned, it has been reported previously that hypothermia causes an inhibition of complement activation and platelet degranulation. Experimental studies[19] have further shown that hypothermia decreases the capacity of serum to generate C3a and C4a in response to a known complement activator (zymosan), with a reversible defect in platelet function. Our investigation is in agreement with this results, because we have found a consistent reduction, although not significant, in plasma levels of C3a and TGF-β1 during hypothermic CPB in comparison to the normothermic technique. This thermic effect is particularly evident in platelet activation and degranulation which slightly affected the C3a production. Further studies are needed to clarify the optimal temperature for the preservation of the circulation homeostasis.

In conclusion, our study suggests that the increased levels of plasma C3a and TGF-β1 seem temporarily related to the inhibition of T-cell proliferation observed during CPB. Because the immunosuppressive effects of these molecules are reported for *in vitro* and *in vivo* studies, we can speculate that these mediators as well as others not evaluated here, are responsible for the immunodepression observed in cardiac patients undergoing CPB.

A final comment concerns the pleiotropic activity of TGF-β1[22] so that it can exert either protective or damaging effects during CPB. As an example, TGF-β1 may be associated with the thermotolerance. Thermotolerance can generally be defined as the ability of an organism to withstand an otherwise lethal stress due to pretreatment with a nonlethal stress. The stress could result from a number of insults such as hyperthermia and oxidative injury which can induce expression of heat stress proteins (HSP) as an acute response. Furthermore, the well recognized role of TGF-β1 as a mediator in tissue repair suggests that its increased generation after the perturbation of cellular homeostasis could be beneficial in promoting recovery of cells and healing.[22] Thus we are now planning to evaluate an immunoadjuvating treatment carried out before and after CPB to prevent or reduce the immunosuppression that may favour septic complications after surgery.

### References

1. Nilsson L, Brunnkvist S, Nilsson U, *et al.* Activation of inflammatory systems during cardiopulmonary bypass. *Scand J Thorac Cardiovasc Surg* 1988; **22:** 51–57.
2. Kirklin JK, Westaby S, Blackstone EH, Kirklin JW, Chenoweth DE, Pacifico AD. Complement and the damaging effects of cardiopulmonary bypass. *J Thorac Cardiovasc Surg* 1983; **86:** 845–857.
3. Chenoweth DE, Cooper SW, Hugli TE, Stewart RW, Blackstone EH, Kirklin JW. Complement activation during cardiopulmonary bypass: evidence for generation of C3a and C5a anaphylatoxins. *N Engl J Med* 1981; **304:** 497–502.
4. Ide H, Kakiuchi T, Furuta N, *et al.* The effect of cardiopulmonary bypass on T cells and their subpopulations. *Ann Thorac Surg* 1987; **44:** 277–282.
5. Hisatomi K, Isomura T, Kawara T, *et al.* Changes in lymphocyte subsets, mitogen responsiveness, and interleukin-2 production after cardiac operations. *J Thorac Cardiovasc Surg* 1989; **98:** 580–591.
6. Slade MS, Simmons RL, Yunis E, Greenberg LJ. Immunodepression after major surgery in normal patients. *Surgery* 1975; **78:** 363–372.
7. Naldini A, Borrelli E, Cesari S, Giomarelli P, Toscano M. *In vitro* cytokine production and T-cell proliferation in patients undergoing cardiopulmonary by-pass. *Cytokine* 1995; **7:** 165–170.
8. Erdei A, Fust G, Gergely J. The role of C3 in the immune response. *Immunol Today* 1991; **12:** 332–337.
9. Roberts AB, Sporn MB. Physiological actions and clinical applications of transforming growth factor-beta (TGF-beta). *Growth Factors* 1993; **8:** 1.
10. Kasid A, Bell GI, Director EP. Effects of transforming growth factor-beta on human lymphokine-activated killer cell precursors: autocrine inhibition of cellular proliferation and differentiation to immune killer cells. *J Immunol* 1988; **141:** 690–698.
11. Lichtenstein SV, Ashe KA, El Dalati H, Cusimano RJ, Panos A, Slutsky AS. Warm heart surgery. *J Thorac Cardiovasc Surg* 1991; **101:** 269–274.
12. Buckberg GD. Strategies and logic of cardioplegic delivery to prevent, avoid, and reverse ischemic and reperfusion damage. *J Thorac Cardiovasc Surg* 1986; **93:** 127–134.
13. Lichtenstein SV, Abel JG, Panos A, Slutsky AS, Salerno TA. Warm heart surgery: experience with long cross-clamp times. *Ann Thorac Surg* 1991; **52:** 1009–1015.
14. Hugli TE, Chenoweth DE. Biological active peptides of complement techniques and significance of C3a and C5a measurements. In: Nakamura RM, Dito WR, Tucker III ES, eds. *Clinical Laboratory Techniques for the 1980s.* New York: Alan R. Liss, 1980; 443–460.
15. Lawrence DA, Pircher R, Kryceve-Martinerie C, Jullien P. Normal embryo fibroblasts release transforming growth factors in a latent form. *J Cell Physiol* 1984; **121:** 184–188.
16. Mosmann T. Rapid colorimetric assay for cellular growth and survival: application to proliferation and cytotoxicity assays. *J Immunol Method* 1983; **65:** 55–63.
17. Brody JI, Pickering NJ, Fink GB, Behr ED. Altered lymphocyte subsets during cardiopulmonary bypass. *Am J Clin Pathol* 1987; **87:** 626–628.
18. Hammerschmidt DE, Stroncek DF, Bowers TK. Complement activation and neutropenia during cardiopulmonary bypass. *J Thorac Cardiovasc Surg* 1981; **81:** 370–377.
19. Moore FD, Warner KG, Assousa S, Valeri CR, Khuri SF. The effects of complement activation during cardiopulmonary bypass. *Ann Surg* 1988; **208:** 95–103.
20. Wahl SM, Hunt DA, Wong HL, *et al.* Transforming growth factor-β is a potent immunosuppressive agent that inhibits IL-1-dependent lymphocyte proliferation. *J Immunol* 1988; **140:** 3026–3032.
21. Mule JJ, Schwarz SL, Roberts AB, Sporn MB, Rosenberg SA. Transforming growth factor-beta inhibits the *in vitro* generation of lymphokine-activated killer cells and cytotoxic cells. *Cancer Immunol Immunother* 1988; **26:** 95–99.
22. Wahl SM. Transforming growth factor β: the good, the bad, and the ugly. *J Exp Med* 1994; **180:** 1587–1591.

ACKNOWLEDGEMENTS. The authors are very grateful to Mrs Daniela Contorni, Debora Castellani, Sonia Muricci and Mr Paolo Migliorini (perfusionists staff) for the expert technical support, and to Miss Patrizia Marrocchesi for her skill in preparing the manuscript. The study has been partially supported by 40% and 60% Grants, Ministry of University and Scientific and Technologic Research (MURST).

**Received 25 September 1995;**
**accepted in revised form 8 December 1995**
